# A Real-Time Web of Things Framework with Customizable Openness Considering Legacy Devices

**DOI:** 10.3390/s16101596

**Published:** 2016-09-28

**Authors:** Shuai Zhao, Le Yu, Bo Cheng

**Affiliations:** 1 State Key Laboratory of Networking and Switching Technology, Beijing University of Posts and Telecommunications, Beijing 100876, China; chengbo@bupt.edu.cn; 2 China Mobile Information Security Center, Beijing 100033, China; yule@chinamobile.com

**Keywords:** Web of Things, devices access, real-time interaction, resource platform, semantic model

## Abstract

With the development of the Internet of Things (IoT), resources and applications based on it have emerged on a large scale. However, most efforts are “silo” solutions where devices and applications are tightly coupled. Infrastructures are needed to connect sensors to the Internet, open up and break the current application silos and move to a horizontal application mode. Based on the concept of Web of Things (WoT), many infrastructures have been proposed to integrate the physical world with the Web. However, issues such as no real-time guarantee, lack of fine-grained control of data, and the absence of explicit solutions for integrating heterogeneous legacy devices, hinder their widespread and practical use. To address these issues, this paper proposes a WoT resource framework that provides the infrastructures for the customizable openness and sharing of users’ data and resources under the premise of ensuring the real-time behavior of their own applications. The proposed framework is validated by actual systems and experimental evaluations.

## 1. Introduction

With the development of the Internet of Things (IoT), resources and applications on top of it have emerged on a large scale. However, most efforts have focused on single applications and “silo” solutions in which devices and applications are tightly coupled. These solutions are characterized by “proprietary devices for one specific application”. Moreover, the market is currently quite fragmented. Each industry vertical has developed its own technical solutions without much regard for reuse and commonality [1,2,3]. Due to the lack of suitable infrastructures and the limited understanding of the two worlds (cyberspace and physically embedded), developers have to bridge this gap “manually” and have to become experts in both worlds. The development of IoT applications is thus cumbersome and inefficient [2,4]. In addition, the multitude of legacy sensor devices will play an important role in the IoT. Consequently, infrastructures are needed to connect sensors to the Internet and publish their output in well-understood, machine-processible formats on the Web thus making them accessible and usable at large scale under controlled access [3]. They should open up or break the current application silos and move to a horizontal application mode [5]. The success of the Web has encouraged researchers to consider incorporating real world objects into the World Wide Web, namely the Web of Things (WoT). In the WoT, each physical object is represented as a Web resource, which can be invoked through a Uniform Resource Identifier (URI) using Web APIs. Based on the WoT concept, many infrastructures have been proposed to integrate the physical world with the Web. However, issues such as no real-time guarantee, lack of flexible and fine-grained control of data and the absence of explicit solutions for integrating heterogeneous legacy devices, hinder their widespread and practical use.

To address these issues, this paper proposes a multi-level WoT framework that includes a public platform (PP) and a local platform (LP). The PP enables applications to reuse and share cross-domain resources, while the LP overcomes the technology heterogeneity within an application domain, and it provides a real-time resource platform for applications within the domain. Specifically, this paper makes the following contributions:

(1) Unified device access (UDA) is implemented based on the Open Service Gateway Initiative (OSGi) and dependency-inversion pattern. It shields the heterogeneity of devices, and then exposes them in a uniform resource way.
(2) A multilevel and multidimensional (MM) model is proposed to describe the resources and information of the framework. It decouples the upper applications from the underneath devices. Based on it, sensing data can be promoted and interpreted to context information. Besides, with the help of data manipulation language (DML), the opening granularity and the aggregation method of sensing data can be flexibly constrained.
(3) The proposed multi-level framework enables the customizable opening and sharing of user’s resources, meanwhile, ensures the real-time of their local applications. Its practicability is validated by actual systems and experimental evaluations.

The remainder of the paper is organized as follows: Section 2 reviews related work and challenges. Section 3 presents the overview of the proposed multi-level framework. Section 4, Section 5 and Section 6 describe the key technologies of the framework. Section 7 demonstrates the practicability of the proposed framework by case study and experimental evaluations. Section 8 concludes the paper and discusses our future work.

## 2. Related Work and Challenges

Based on the concept of WoT, many commercial platforms have been proposed, such as Cosm [6], SenseWeb [7], GSN [8], and Yeelink [9], etc. These infrastructures act as data platforms, which manage millions of data points from thousands of individuals and companies around the world. They enable people to share, discover and monitor sensor and environmental data from objects that are connected to the Web [2,10,11]. However, these existing efforts are still far from forming an ecosystem. They lack commercial and practical applications; most resources are provided by enthusiasts; the applications on top of them are still prototype or “toy level”.

Apart from these commercial platforms, multiple research projects (such as PECES, SemsorGrid4Env, SENSEI, and IoT-A), aiming to propose solutions for the integration of the physical world with the cyberspace, have been launched. The PECES architecture [12] provides a software layer to enable the seamless cooperation of embedded devices across various smart spaces on a global scale. It enables dynamic group-based communication between PECES applications by utilizing contextual information based on a flexible context ontology. Entities are encapsulated as contextual information, a model abstraction which can include spatial elements, personal elements and devices and their profiles. The PECES Registry implements a Yellow Pages directory service. Any service matching that description may be returned by the registry. The SemSorGrid4Env architecture [13] provides support for the discovery and use of sensor-based, streaming and static data sources. The architecture may be applied to almost any type of real world entity, although it has been used mainly with entities related to natural phenomena. The types of resources considered are: sensor networks, off-the-shelf mote-based networks or ad-hoc sensors, and streaming data. These resources are made available through a number of data-focused services (resource endpoints), which are based on the Web Services Data Access and Integration (WS-DAI) specification for data access and integration.

The SENSEI architecture [14] aims at integrating geo-graphically dispersed and internet interconnected heterogeneous Wireless Sensor and Actuator Networks (WSAN) systems into a homogeneous fabric for real world information and interaction. In the SENSEI architecture, each real world resource is described by a uniform resource description, providing basic and semantically expressed advanced operations of a resource, describing its capabilities and REP information. On top of this unifying framework SENSEI builds a context framework, with a 3 layer information model. One of the key support services is a rendezvous mechanism that allows resource users to discover and query resources that fulfill their interaction requirements. The IoT-A project [15,16] extends the concepts developed in SENSEI further to provide a unified architecture for IoT. It aims at the creation of a common architectural framework for making a diversity of real world information sources such as wireless sensor networks accessible on a Future Internet. Although lots of significant works have been proposed, some challenges which hinder the widespread use and further development of IoT still exist:

(1) Real-time and reliability. IoT application scenarios are time-critical and reliability-critical [5], the premise of resource sharing by vendors or developers is ensuring the real-time and reliability of their own applications. But these platforms cannot guarantee this premise. The delay of data uploading and getting is uncontrollable. If commercial users open their resources and develop applications on top of them, they have to bear two times network delay (data from devices to remote platform and from remote platform to applications).
(2) Customizable Openness. Users need flexible and fine-grained control on how much information will be published (e.g., direct access, regular summary); how their sensor data are shared (e.g., read-only, time-delayed) and with whom (owner-only, specific individuals) [4]. But existing platforms lack the mechanism for users to control their data. For example, Cosm only provides two options (private and public), if a data stream is set to public, all data of it is exposed to anyone [1].
(3) Legacy devices. These platforms focus on the solution of accessing (plugging) new devices, some of them are only able to access their customized devices. They lack the explicit solution for the integration of legacy devices which are important but quite fragmented and heterogeneous. A unified solution is required to integrate heterogeneous devices and expose their capabilities in uniform interfaces.
(4) Resource and information model. Resource and information model is the foundation of essential platform services, such as resource selection and data interpretation [2,16]. Existing commercial platforms (such Cosm, SenseWeb) lack of such model, their data lie on the raw data or observation data. However, users are interested in real-world entities and their high-level states. Although research projects (e.g., SENSEI, IoT-A) have proposed meaningful models for modeling resource, entity and their mapping relationship, these models tend to mix resources’ multiple feature dimensions to construct an integrated ontology hierarchy. This dimension-mixed way is difficult to build a well-defined resource hierarchy, and may not be able to select resource and establish resource-entity mapping automatically.

## 3. Framework Overview

The premise of resource sharing is ensuring the real-time and reliability of resource providers’ applications. Although existing platforms like Cosm and io.bridge have proposed several exchange modes (e.g., MQTT and Pawl) to improve their interaction efficiency, the real-time and reliability still cannot be guaranteed. The data exchange delay is uncontrollable and unpredictable, as it depends on network conditions. If commercial users open their resources and develop applications on top of them, they have to bear twice the network delay. Moreover, these existing platforms limit the fastest frequency of data exchange. If users make more frequently request than it allows, then none of their requests will receive a response. For example, the Yeelink platform sets the minimum time interval between two Hypertext Transfer Protocol (HTTP) requests regarding the same data as 10 s. If a user requests the data too frequently, the platform will not answer his requests and return a “403 error” [10].

**Figure 1:**
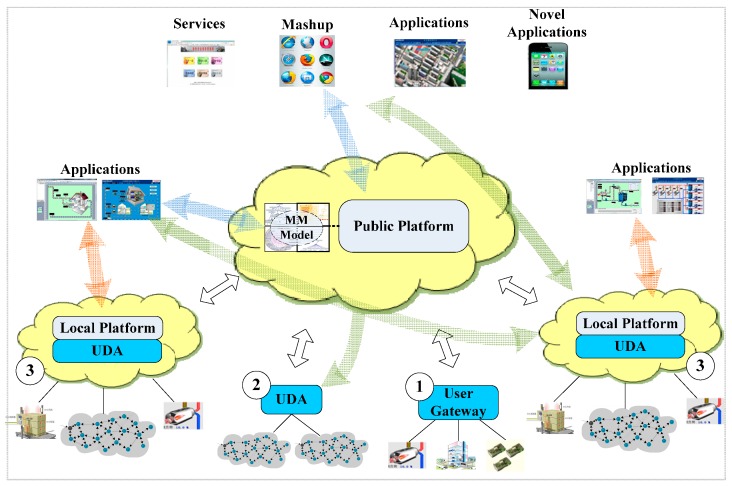
Framework Overview.

As shown in Figure 1, we propose a multi-level framework that provides resource openness, while avoiding the unnecessary delays and instability caused by such openness. It consists of a public platform (PP) and a local platform (LP). The PP enables users to share sensor data from objects that are connected to the Internet. It provides lightweight interfaces to facilitate the composition of new services that incorporate devices from different domains. Applications can interact with each other, and their data and services can be integrated with resources in other systems. Any terminals that support the Internet can access these resources rather than customized clients. Moreover, it reduces costs by reusing common resources (e.g., storage resources and communication resources like SMS).

Many IoT applications are time-critical and reliability-critical [5], such as a coal mine monitor systems for monitoring harmful gases. Details of this system will be presented in Section 7.1. Moreover, even within a single organization or domain, the number of heterogeneous solutions can be large. These solutions provided by different hardware or software manufacturers are isolated from each other. The resources and data are locked in each system, and cannot be shared and reused. For example, in the coal mine scenario discussed in Section 7.1, the monitoring system is isolated from the reporting system because they were developed by different manufacturers. Hence, users have to copy the data from the monitoring system, perform calculations, and fill in the reports manually. Thus, in addition to the PP which opens resources, the LPs, which provide real-time and reliable services for applications, are necessary. The LP also acts as an infrastructure for inner-domain resources sharing and reuse. It maintains the extensibility of systems in the domain, and new devices and applications can be easily integrated into it. Meanwhile, existing applications can be extended agilely to reuse devices and interact with other applications. Therefore, the LP is very useful for commercial application scenarios even if they do not want to open their resources. The framework proposes three approaches for accessing (plugging) new or legacy devices, as demonstrated in Figure 1:

(1) The individual users that just want to access their devices in a simple way just need to create their resources on the PP and copy a piece of common code to their gateways (e.g., an Arduino board or PC), and then fill in the URI of the resources they created. After that, the data can be uploaded or retrieved through the PP using the resource’s URI.
(2) If the resource providers want to access multiple heterogeneous devices, they can use the UDA to shield the heterogeneity of devices without interfering with the legacy Wireless Sensor Networks (WSNs). The UDA will expose service endpoints directly or upload well-formed data to the PP.
(3) Commercial users can use the UDA to shield the heterogeneity of devices. Then, the LPs expose the resource capabilities as light-weighted services and publish the resources that the users want to share to the PP.

After that, users can open the resource, meanwhile, flexibly configure the degree of openness using the DML. For example, they can configure whether others can direct access their LPs or only access the data uploaded to the PP. They can also configure to share the aggregation or summary of data periodically, rather than sharing the entire data stream. Moreover, the framework provides multiple interaction modes according to the degree of resource openness:

(1) Centralized mode: As indicated by the thick blue lines of Figure 1, consumer applications retrieve data from the PP, which collects data from distributed devices, using push-based (publish/subscribe) or pull-based (request/response) methods. The PP acts as a data broker center which is responsible for all data uploading and retrieving.
(2) Peer-to-Peer mode: This mode is indicated by thick green lines. It is a distributed “peer-to-peer” interaction mode in which resource consumers directly access resources from the LPs (or UDA) of other domains. The LPs expose their resources in Restful interfaces. Consumers can invoke their resources using standard URI, and this mode avoids the delay from the devices to the public platform. In this mode, the PP acts as a directory or rendezvous of resources, which provides the discovery and selection services for users to obtain the access address of their desired resources.
(3) Local mode: Demonstrated by thick red lines, the applications of resource providers invoke their own resources on top of the LP within an application domain. The interaction of this mode may be within an intranet, which avoids the delay or unreliability of external network. The LP provides real-time and extensibility services for applications within the domain. These applications can also use resources provided by other application domains using the above two modes.

**Figure 2:**
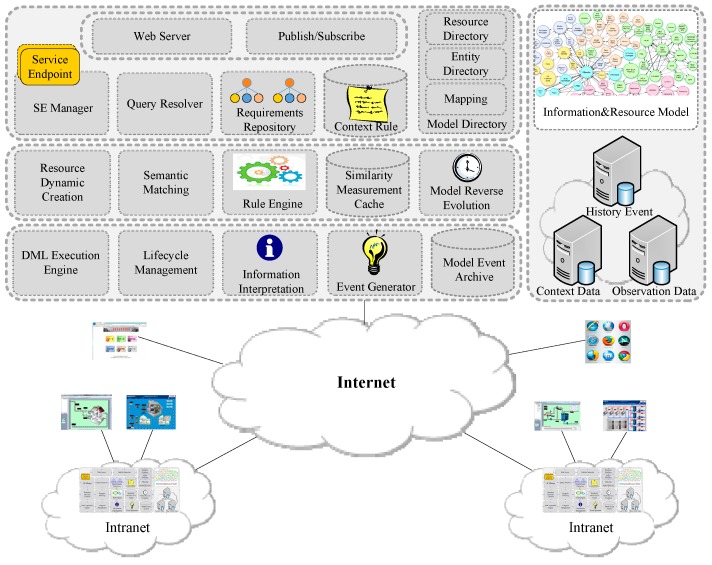
Internal architecture of resource platform.

Figure 2 shows the internal architecture of the resource open platform. When a new resource accesses it, its resource description is published to the platform, which will be maintained in resource model. The Event Generator generates a new resource accessing model event and sends it to the Model Event Archive. We call the relationship that a resource provides services (observe or control) for an entity as resource-entity binding. For each request, the platform uses the Model Directory to find out which resources can provide information for the requested entity attributes. If no resource is associated to an entity attribute, the framework first tries to use available context information and resource descriptions to establish new associations. The Query Resolver analyzes and transfers the requested resource descriptions to an abstract resource description (ARD). The Semantic Matching calculates the matching degree based on the entity and resource model. Then it recommends the matching resources to the user. User chooses the desired resource, and then this resource will be bound to the entity which is needed to monitor. The binding has two meanings: when users want to observe or change the state of entities, the platform can invoke corresponding resource services; observation data can be interpreted to context data and events. The UDA transfers the heterogeneous raw data into the resource-level observation data, and then the Information Interpretation promotes the observation data to the context data based on the entity model and resource-entity binding. Next, the Event Generator will generate events if the data match the event conditions. The same observation data can be interpreted to multiple meanings according to the entity model which represents the context of the application scenario.

## 4. Unified Device Access

Existing platforms like Cosm and io.bridege focus on the solutions for accessing new devices. However, they lack a well-defined solution for the integrating legacy devices which play an important role but are quite fragmented and heterogeneous. A device adaptation mechanism is required to access these devices and normalize the way they are exposed via a unified API to applications, and it should have an application-independent way of describing the capabilities regardless of whether it is a ZigBee or Z-Wave device. That is exactly what the UDA has in place.

**Figure 3:**
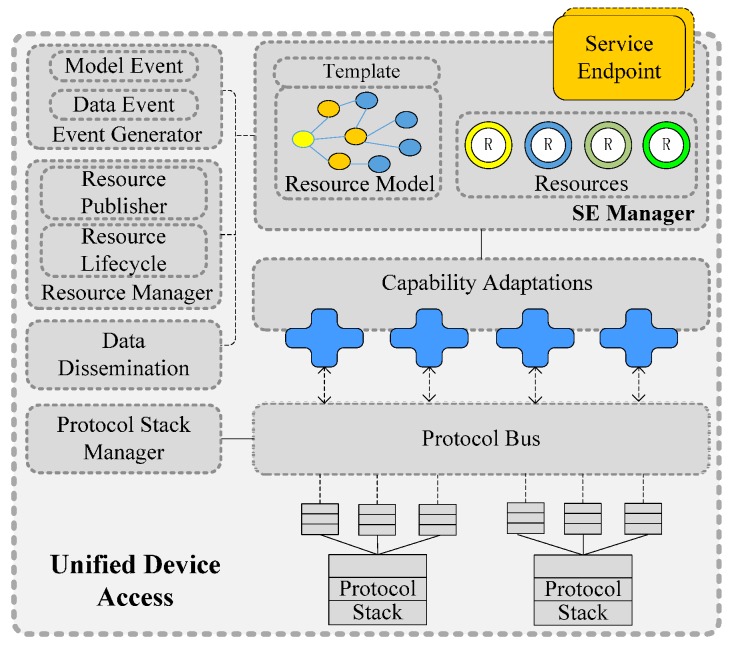
Internal structure of UDA.

**Figure 4:**
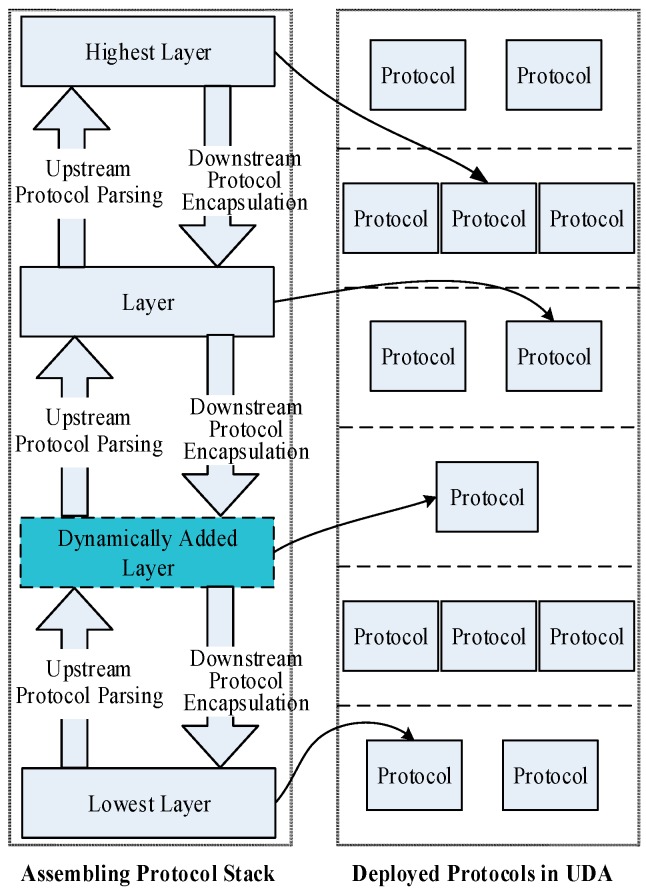
The dynamic assembling of protocol stack.

As shown in Figure 3, it can integrate heterogeneous devices on one side and expose uniform service interfaces on the other side. The responsibility of device access includes not only adapting the protocols but also adapting the device capabilities, publishing the resources, and following up resource lifecycle management, etc. UDA has the following characteristics:

(1) The protocol stack is plug and play (PnP); based on the dynamics of OSGi and the dependency-inversion pattern, UDA is flexible and High Cohesion and Low Coupling (HCLC) device protocols within UDA are encapsulated as standard OSGi bundles. Protocols can be deployed or re-deployed dynamically. Moreover, protocol stacks can also be assembled and re-assembled dynamically, as shown in Figure 4. After a new protocol is deployed from a compiled jar file or a new protocol stack is assembled by several protocols, it registers itself to the protocol stack manager. Then this new protocol stack will immediately work to parse real-time packets without restarting the program. The entire process is PnP without influencing of the adaptation continuity of other protocols.
(2) Heterogeneous protocols can be adapted automatically. When a new device accesses, the device packet is published on the protocol bus. Then, every protocol stack will check whether it can correctly parse the packet. If a protocol stack is able to parse the packet, it will respond to the protocol stack manager. The protocol stack manager binds the device with an instance of this protocol stack using an identifier (such as TCP/UDP port number) that can distinguish different devices and is accessible by the protocol stack. Then, packets provided by this device are handed over to this instance for parsing.

**Figure 5:**
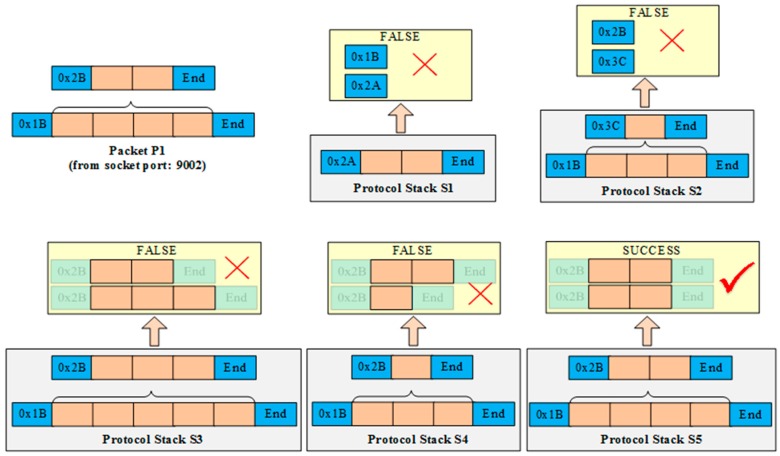
An example of judging which protocol stack could correctly parse a new packet.

Figure 5 shows a simplified example of selecting a suitable protocol stack to parse the packet P1 correctly. Each small block represents a byte length of the datagram. An orange block denotes the data payload part of the datagram, and a blue block denotes the metadata part of the datagram, which includes the header, tail, length, Cyclic Redundancy Check (CRC), etc. Assuming that, a new packet P1 is transmitted from port 9002 using a socket connection, and five protocol stacks (S1–S5) are assembled in the UDA. P1 is encapsulated by two protocols, the header of the underlying protocol is 0 × 1 B and the header of the upper protocol is 0 × 2 B. When P1 arrives, it is published to every protocol stack concurrently, then each protocol stack tries to parse P1 using its protocols. The yellow boxes stand for the parsing results of every protocol stack. S1 fails to parse P1 because S1’s protocol header (0 × 2 A) does not match with P1’s header (0 × 1B). The header and tail of S2’s underlying protocol can match with P1, then it uses its protocol to parse P1 and extracts the data payload which is the complete packet of the upper protocol of S2. Next, S2 uses its upper protocol to parse the packet extracted by the previous step, and the result is false because the header (0 × 3C) of S2’s upper protocol does not match with P1’s upper protocol (0 × 2B).

S3 uses its underlying and upper protocols to parse P1. The headers of S3 are consistent with P1 but S3 still cannot parse P1 correctly because P1’s packet length is shorter than S3’s protocol format. Similarly, S4 also fails to parse P1 because the length of the result that using S4 to parse P1 is longer than S4’s protocol format. Finally, only S5 is able to parse P1 correctly because the packet format and packet length of every protocol (from bottom to top) are consistent. Then S5 parses and extracts the data payload of P1, and translates it into application-available numerical values. In addition, the protocol stack manager binds S5 with the identifier that can recognize the source of P1. In this example, it binds socket port 9002 to S5. Subsequently, all packets from port 9002 are pushed to S5 directly.

If no protocol stacks can parse this packet, the UDA tries to dynamically assemble a protocol stack based on the deployed protocols. As demonstrated in Algorithm 1, the deployed protocols are divided to different layers. The protocol stacks are consisted of these deployed protocols (illustrated in Figure 4). The protocol stack manager publishes this raw packet to the deployed protocols. If a protocol can parse the outermost layer protocol, it becomes the lowest layer of the new protocol stack, and then it parses the raw packet and extracts the data payload part. Sequentially, the data part is published to other deployed protocols that lie in upper layers. At last, the other layers and the highest layer of the new protocol stack can be assembled by the similar process. If no suitable protocol stack can be assembled, the framework returns the failure result which means that existing deployed protocols cannot parse this packet and new protocols are required. The process is automatic without human intervention.

**Algorithm 1.** Automatic protocol adaptation.**Input:**  unknown packet: *packet* **Output:** matched protocol stack: *ps* **Initialize:** *PS*={*ps1,ps2,…,psi,…,pss*} //existing protocols stacks     *PC*={*layer_1_,layer_2_,…,layer_i_,…,layer_l_*} //protocol container     *Layer* = {*p_i1_,p_i2_,…,p_ij_,…,pi_p_*} //deployed protocol in layer i     SendToBus(*packet*); //send packet to protocol bus   **for all protocol stack**
*ps* ∈ *PS*
**do**     if (*ps_i_*.PsAnalyze(*packet*)==1){Return *ps_i_*;} **end for**     Ps *ps*=new PS();   **for all protocol**
*p* ∈ *layer1*
**do**     boolean *b*=TryAnalyze(*packet,ps,p,layer_1_*);     if (*b*==false){Return Null;}     Return *ps*; **end for** **Procedure** TryAnalyze(*packet,ps,p,layer*) //p is located in layer    *payload* =*p*.Analyze(*packet*);    If (*payload*==0){      *ps*.push(*p*);      Return true;}    *layer_i_* = *layer*.next();    If (*layer_i_* == null){Return false;}   **For all protocol**
*p_i_* ∈ *layer_i_*
**do**    boolean *b* = TryAnalyze(*payload,ps,p_i_,layer_i_*);    if (*b* == true){ *ps*.push(*p*);}    Return *b*; **end for** **Procedure End**

Device identification and information can be extracted from the access packet. The Service Endpoint (SE) Manager initializes a resource instance and assigns a resource URI for the device according to the corresponding resource template. This URI is the identification and the access address of resources. Then it puts the extracted device information into the resource description, it is well-defined according to resource model. After that, “southbound” heterogeneous devices are exposed “northbound” in a uniform resource way. A device can provide one or several resources, for example a sensor node may provide temperature and humidity data, then it can be exposed as temperature sensing and humidity sensing two resources. In this case, the resource need to bind with the two level identifications of device, for example device ID plus the I/O channel number of each sensor or Programmable Logic Controller (PLC) number plus register address.

However, the resource instance is not yet complete because the auto-generated information is limited. Then the device providers should further describe it, and attach some metadata or semantic information which is used for following resource discovery and data interpretation. Based on multiple dimensional model described in Section 5, resources are further described with the help of resource modeling tool which is developed by us based on OWL-API [17] and Java SWT [18]. By modeling tool, the information of every feature dimension is described, includes the inheritance hierarchy of resource class, class axioms (subclass, equivalent, disjoint), class description (intersection, union and complement), resource’s property (data property and object property), resource relationship, restriction (value constraints, cardinality constraints), etc. The detail of modeling tool is out of the scope of this paper, and will be discussed in our future works. After that, the resource’s information of every dimension is described, such as *measurement principle dimension, measure quantity type, operating and survival range, spatial dimension*, and so on. The UDA publishes the described resource to the platform, and then the resource publication process is finished.

When the data packet that is provided by the device arrives, the protocol stack parses it and extracts the data part, and then processes it to form the unified-format raw data according to its offset and factor. The raw data are the lowest layer of the information model and contains the value observed by the device. It can be enhanced with metadata, e.g., units, the observed resource and QoI (Quality of Information) described in the resource model. Then the device-level raw data are promoted to resource-level observation. Moreover, if the data match an event condition, the Event Generator creates and publishes the corresponding data event. The Resource Manager monitors the lifecycle state of resources. The resource is periodically synchronized with the actual state of the physical device via its protocol stack. The state information is represented as a model event. A “resource failure” event will be caused by the failure of the device.

## 5. Multilevel and Multidimensional Model

The information provided by resources ranges from the raw data, to observation data containing meta-information, to the high-level context information required by specific applications and services [19]. Given the heterogeneity and fragmentation of the IoT environment, a well-defined resource model is necessary to shield the heterogeneity of device descriptions and represent them in a unified resource form. The MM model is proposed to decouple the upper applications from the lower devices. It defines the characteristics and properties of the resources, which include the information such as the physical properties being observed, the properties of the resource’s measurement capability; the physical structure on which the resource is deployed and its location; and the environmental conditions under which the resource is expected to operate. By association of resources and context, data can be interpreted to context information, and application-related meaning can be extracted within the context.

**Figure 6:**
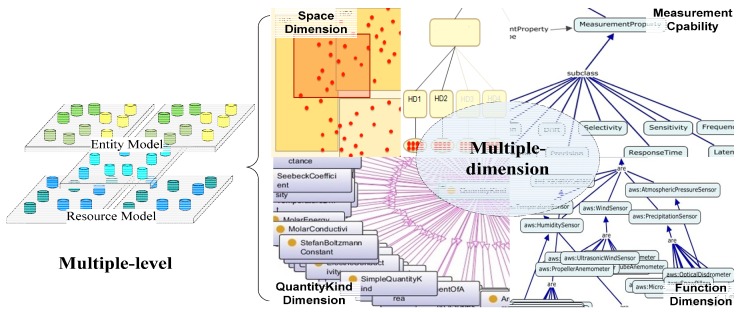
Multilevel and multidimensional model.

As shown in Figure 6, one characteristic of the model is that it is multilevel. It decouples the resource model from the entity model, which enables the decoupling of applications from the devices from the data model’s viewpoint. Application developers only need to concentrate on the entities and application logic, and describe the entities and domain knowledge. The entity model contains the properties of the entities and their relationships. Moreover, different applications can interact with each other by linking their entities in respective entity model. Meanwhile, resource providers only need to describe their resources. Therefore, the entity model of an application scenario can be related to multiple resource models, it means that applications can share resources at the model level. However, neither independent level is sufficient for resource selection and data interpretation, it needs both resource and entity information. The platform combines multilevel information using Linked Data and common ontology, such as Quantities and Units (QU) ontology [20] and Climate and Forecast (CF) features [21] ontology.

Multi-dimension is another characteristic of the model. We extract critical feature dimensions of resources to construct multidimensional models. Each dimension constructs a model independently which includes the taxonomy, property, description and restrictions of this dimension. For example, the dimensions of *measurement principle*, *measure quantity type*, *measure capability*, *operating and survival range*, and *spatial* are extracted to construct the resource model.

The *measurement principle* indicates the physical effects that can be used to convert stimuli into electric signals. For example, the measurement principle of capacitive water-level sensor is dielectric-constant. According to the measurement principle, users can select the appropriate resources that are more suitable for their application context. For example, magnetic sensors (according to magnetism) are often used to detect motion, displacement, position, and so forth. But they are unsuitable for a magnetic interference scenario. *Quantity* *type* indicates the physical quantity that the sensor observes. For example, an air-filled capacitor may serve as a relative humidity sensor because moisture in the atmosphere changes air electrical permittivity. Thus, the quantity type is atmosphere humidity. The quantity type model is built based on the W3C CF-feature ontology, which is an ontology representation of the generic features defined by Climate and Forecast standard vocabulary.

The *measure capability dimension* is the key performance indicator of resources for selection. It consists of concepts such as frequency, accuracy, drift, and sensitivity. As the operation of resources will be affected by the observable conditions, the model describes the measuring capabilities of the resources under certain conditions. It can be used to define how a sensor will perform in a particular context to help characterize the quality of the sensed data. The *operating and survival range dimensions* describe the characteristics of the environment and other conditions in which the sensor is intended to operate. *Operating range* describes the environmental conditions and attributes of a resource’s normal operating environment. *Survival range* describes the environmental conditions wherein a sensor can be exposed without causing damage. *Spatial location* is an indispensable criterion for resource matching and determines whether the location of entity is located in the observation area of resource. The location is defined in terms of the geographical coordinates (hasLatitude, hasLongitude). It also has properties that link to a symbolic location, which has two types: global and local location ontologies. The global location ontology URI links the resource to existing high level location ontologies e.g., GeoNames [22,23], which provides the toponymies or names of cities, districts, countries, etc. The local location ontology provides a detailed location description that captures indoor location concepts representing objects such as buildings, rooms, or other premises.

With the help of the multi-dimensional resource model, the information of plugged resource will be described clearly in each feature dimension, and it enables selecting resource and establishing resource-entity mapping automatically based on based on semantics. The platform selects suitable resources by calculating the semantic similarity between resource and entity (or resource request). It transfers resource requests to structured ARD. ARD includes the monitored entity, properties of it, and the type (mandatory or optional) of every required feature dimension. The semantic matching can accurately measure the similarity of each dimension in parallel. Users can aggregate the measurement values of different dimensions according to their preferences (e.g., set different weights on different dimensions).

After a user selects the most preferred one from the recommended resource list which includes resources with the highest semantic similarity with ARD, the mapping of the monitored entity and the selected resource is built, namely resource-entity binding. Users need only to operate the properties of entity (get/put) to get required data or control their state, while the details of resource invoking are transparent to them. The information of different layers is possibly converted from one to the other through mappings and transformations. The mapping of observation into context information is done explicitly at the time of information delivery by assigning the observation to the attribute value of entity. For instance, the average temperature is an attribute of a room and its value comes from averaging all values of sensors in the room.

## 6. Data Manipulation Language

In order to flexibly configure the degree of openness, we propose DML which is a light-weighted declarative language. Using DML, resource providers can configure the level of opening information (raw data, resource level, entity level), the granularity of opening data (summarized or detail), and the method of data aggregation (operation and time granularity). Moreover, users can dynamically create resource compositions base on existing resources.

Table 1 depicts the grammar of DML. Users can create new resources which act as proxy or aggregation resources for existing resources. Then they use DML to configure the resource content by referencing and aggregating the resource-level (resource observation) or entity-level (entity property) information.

**Figure 7:**
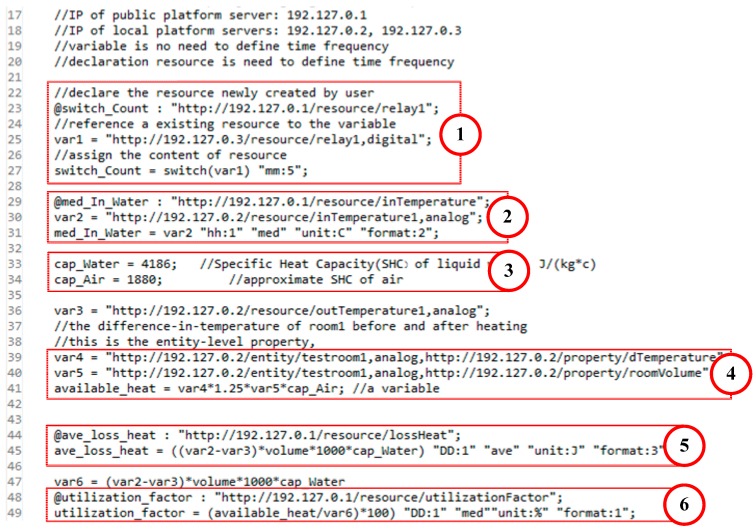
An example of DML from a heating scenario.

Figure 7 shows an example of DML from the scenario of heating discussed in Section 7.1 (case study). For simplicity sake, the calculation formulas are simplified and the IPs are internal addresses for testing. In the example, the IP of PP server is 192.127.0.1; two LPs with the IP: 192.127.0.2 (LP2) and 192.127.0.3 (LP3) respectively. LP2 provides two analog resources:

*inTemperature1 (http://192.127.0.2/resource/inTemperature1)*, observing supply water temperature.
*outTemperature1 (http://192.127.0.2/resource/outTemperature1)*, observing outlet water temperature.

It also provides an entity: *testroom1 (http://192.127.0.2/entity/testroom1)* which represents a monitored room in the heat metering scenario. LP3 provides a digital resource: 10

*relay1 (http://192.127.0.3/resource/relay1)* which indicates the status of relay1.

Users create proxy or aggregation resources on the PP. As shown in number 1 of Figure 7, it declares the resource *switch_Count* to represent for the resource *(http://192.127.0.1/resource/relay1)* newly created on the PP. *switch_Count* is a summary of *relay1*, which counts the number of state switching between close and open every five minutes. 

As shown in number 2, *med_In_Water (http://192.127.0.1/resource/inTemperature)* acts as an aggregation resource for inTemperature1, which gets the median value of inTemperature1 every 1 hour. Its unit is Celsius degree, and the value has two digits after the decimal point.

Number 3 shows the definition of two constants: the specific heat capacity (SHC) of liquid water (*cap_Water*) and the approximate SHC of air (*cap_Air*). Then we reference the properties of *testroom1* entity: *dTemperature* represents the difference-in-temperature of the room after heating; *roomVolume* represents the volume of the room. Then we use them to calculate the available heat used for room heating, which is shown in number 4.

As shown in number 5, by aggregating the observation of *outTemperature1* and *inTemperature1* we can get the average heat loss of supply water, which is declared as:

*ave_loss_heat (http://192.127.0.1/resource/lossHeat)*. Its unit is Joule, and the value has three digits after the decimal point.

As number 6 shows, users can dynamically create a composition resource *(http://192.127.0.1/resource/untilizationFactor)* based on the existing resource and entity information. It is declared as *untilization_factor* representing the effective utilization rate of heat, which is calculated by the ratio of utilization heat to loss heat (supply water).

## 7. Case Study and Evaluation

### 7.1. Case Study

**Figure 8:**
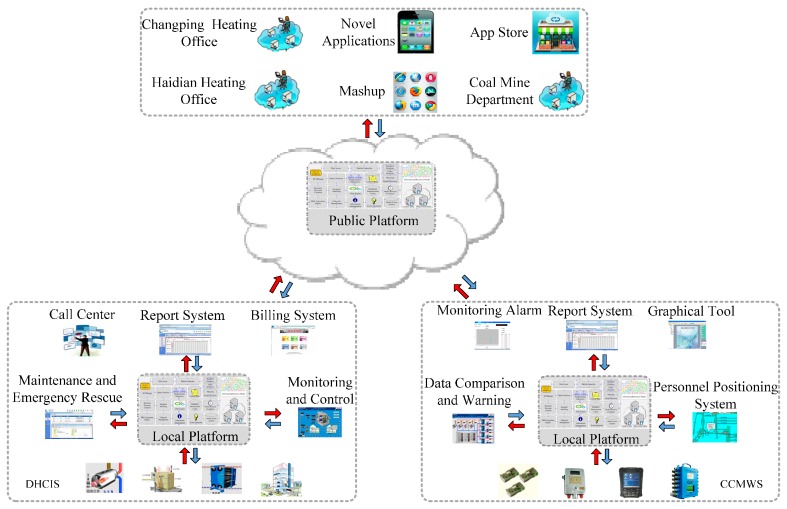
Framework demonstration.

Figure 8 shows the District Heating Control and Information System (DHCIS) and the Coal Mine Comprehensive Monitoring and Early Warning System (CCMWS) developed by us, which are in actual use. In this section, we will use these two application scenarios to demonstrate how the framework works. They access monitoring resources and systems to realize the goal of perception information sharing, comprehensive treatment and analysis, and cross systems service coordination. The DHCIS will cover 120 heating districts and include 200 monitoring boiler rooms and heat transfer stations in Beijing. The perception layer resources include heat metering sensors, flow sensors, pressure sensors, and room temperature detectors. The CCMWS integrates the resources and data of the application domain to ensure the safety of coal mine production and to realize office automation. The devices of these scenarios are fragmented and heterogeneous. The DHCIS scenario has hundreds of devices provided by different manufacturers which support different protocols such as H7000, Modbus, IHDC and M-bus, etc. Initially, the systems of these scenarios are developed by multiple manufacturers and isolated from each other. The resources and data are closed in each system, and cannot be shared. The LPs provide an infrastructure for inner-domain resources sharing and reuse based on resource model and entity model. For example, report system interacts with monitoring system and billing system by temperature and water flow observation resources, heat metering and consumer entities to share data and generate reports.

The PP provides the open platform for cross-domain resource sharing and interaction. For example, by customizable openness, heating offices can supervise the real-time heating situation of the heating companies; coal mine department can monitor the safe condition of mining. Meanwhile, some data can be opened to meet the needs of the public, such as showing the heating data to consumers. With the development of IoT, more and more domains will open their resources and data, then numerous novel applications, DIY apps and app stores, which are based on real-world resources, will emerge.

**Figure 9:**
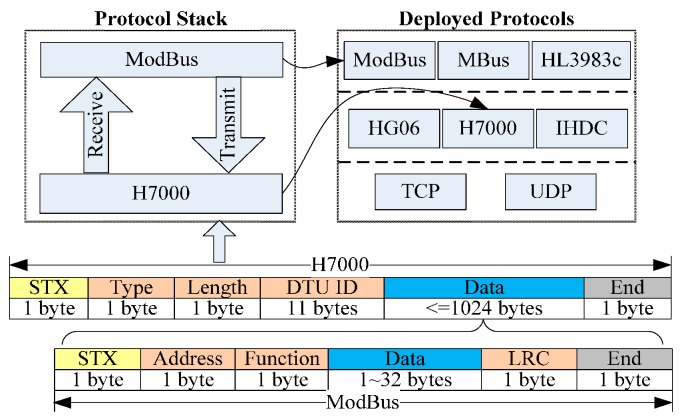
Example of protocol stack.

To demonstrate the framework more intuitively, the following part of this section presents some practical examples of DHCIS. Most of the protocols in DHCIS are industrial control protocols. For simplicity sake, we only present two protocol layers: (data terminal) communications protocol and data protocol. As shown in Figure 9, when a new packet arrives, the protocol manager publishes it to all communication protocols. As the process discussed in Section 4, the H7000 protocol can parse this packet, and then it becomes the lowest layer of the assembling protocol stack. Then it parses the packet and extracts the data part. Sequentially, the data part is published to deployed protocols lie in upper data layers. The ModBus protocol can parse this packet. Then it becomes the highest layer of the protocol stack. At last, the ModBus and H7000 are assembled into the new protocol stack. The protocol stack parses the packet and extracts the device ID, and then the Protocol Stack Manager binds the device with one instance of this protocol stack. After that, packets provided by this device are handed over to this instance to parse.

**Figure 10:**
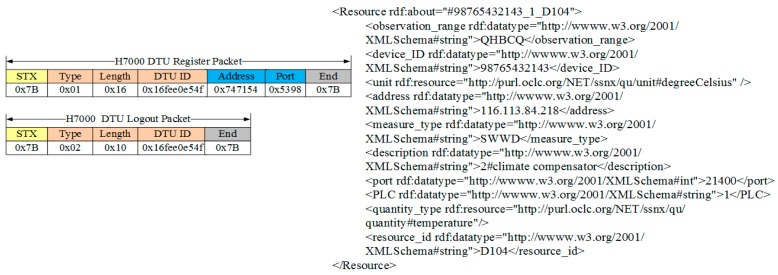
The process of device accessing and resource description.

**Figure 11:**
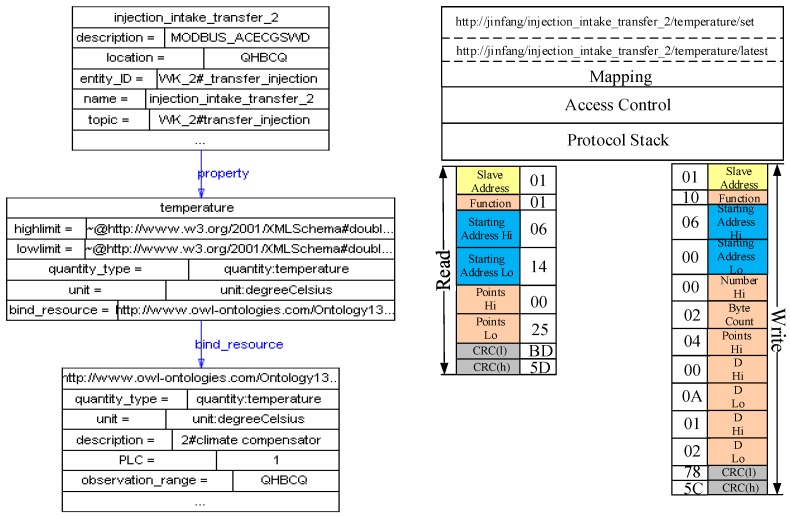
Resource-entity binding and resource operation mapping.

As Figure 10 shows, the data terminal sends a H7000 register packet to the UDA. The protocol stack framework parses this packet. Then, it extracts the device information from the packet and publishes a resource description according to the resource template. As Figure 11 shows, the device provides a temperature resource. The quantity type and unit property are linked to the standard physical quantity of QU ontology. Then, the SE Manager maps the internal operations of the resources to Restful service interfaces. For example, the new temperature observation resource can provide a get sensing data operation, and then SE Manager maps this operation to a get service:

http://jinfang/resource/98765432143_1_D104/realtime/latest

Multi-dimension is another characteristic. We extract some critical feature dimensions of resources to construct multidimensional models. Each dimension constructs a model independently which includes the taxonomy, property, description and restrictions of this dimension. For example, the dimensions of *measurement principle*, *measure quantity type*, *measure capability*, *operating and survival range*, and *spatial* are extracted to construct the resource model. 

**Figure 12:**
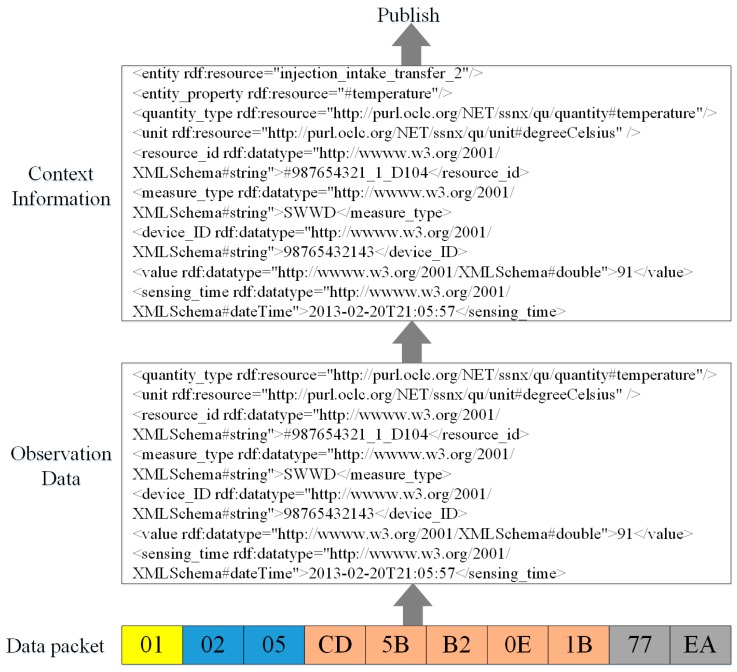
Information publishing process.

Figure 12 shows the sensing information publishing process. When a data packet arrives, the protocol stack parses it and extracts the data part. “5B” stands for the real-time value of resource *1_D104*, and then the protocol stack translates the value to a raw value according to the configured offset and coefficient. Next, the protocol stack combines this value with some meta-data, such as unit and quantity type, and generates the observation data according to the resource model. Then, the Information Interpretation component interprets this observation data to context information according to the resource-entity binding. The same sensing data can be interpreted to different context information according to different domain entities. In this example, the data are interpreted to the *temperature* property of *injection intake* located in *2#transfer station*.

**Figure 13:**
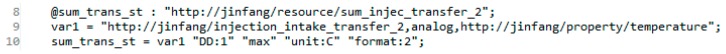
Summary temperature.

As demonstrated in Figure 13, we can configure a resource *sum_trans_st* to expose the temperature property of entity *injection_intake* in an aggregation way. It represents the maximum temperature value of every day, it means that the user only wants to open the summarized data of this property instead of open the detail of every data point of it.

### 7.2. Evaluations

In order to evaluate the performance of the framework, experimental evaluations are necessary. The experiments were carried out on an Intel Core i3-2310M processor 2.10 GHz PC, with 4 GB of RAM running on 32-bit Windows 7 Professional. All servers of LPs and PP have the same specifications.

**Figure 14:**
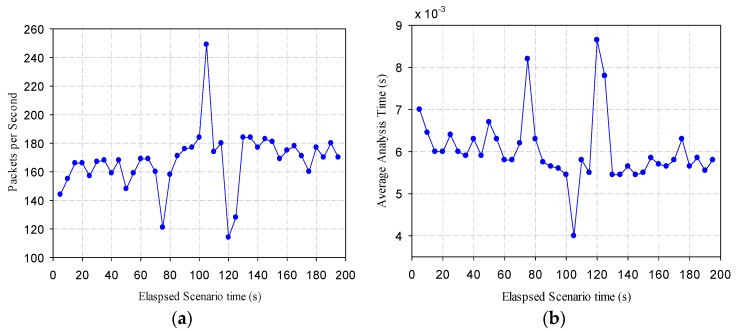
(**a**) parsed packets per second; (**b**) average parsing time of one packet.

UDA is the data source of the platform, so its protocol parsing performance should be evaluated. We simulated 500 data senders to independently send real protocol packets. The protocol stack parses these packets according to the real parsing process. As shown in Figure 14a, the max parsing speed is 250 packets-per-second, the min is 117, and the average parsing speed is 183.5 packets per second. From Figure 14b, we find that the average parsing time of each packet is 5.45 ms.

**Figure 15:**
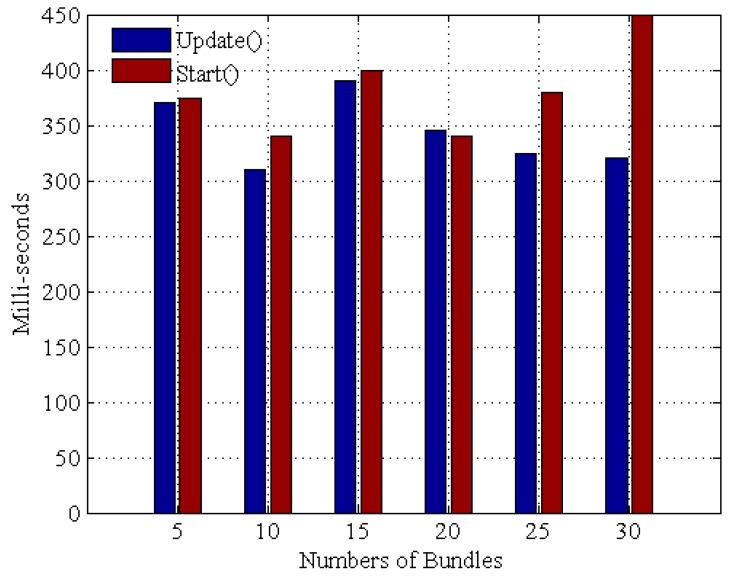
Execution times with bundles variation.

Then, the performance of maintaining the protocol bundle is evaluated, which is measuring the execution time to start and update the protocol bundles of UDA as the number of protocol bundles increases. As Figure 15 shows, when the number of bundles increases, the execution time of the two methods Start() and Update() seems to vary independently of the number of bundles. In addition, we compared the response time for the OSGi framework against a native traditional Java code approach. Specifically, each device protocol bundle is defined as sequentially gathering data, and the data retrieval latency for both approaches is set to 100 ms.

**Figure 16:**
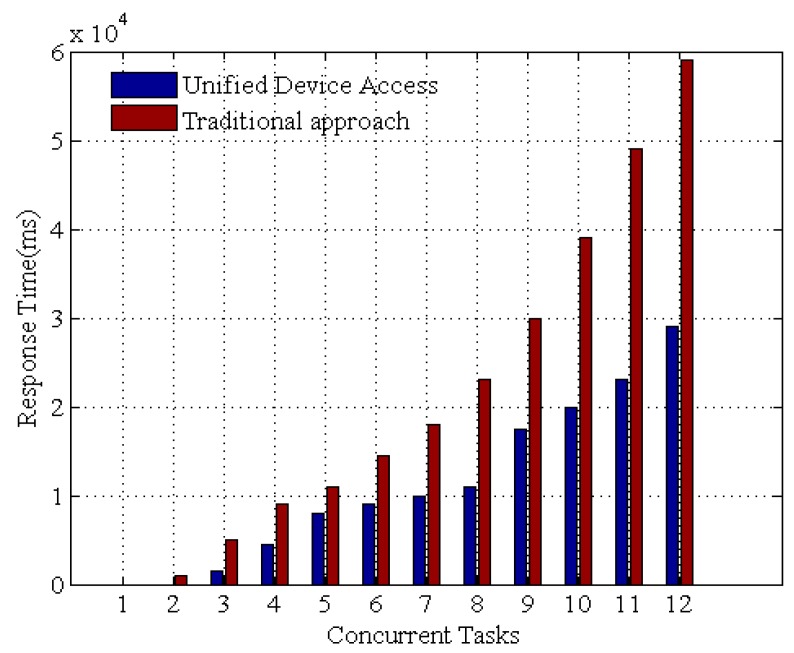
The response time for OSGi framework against native traditional Java code approach.

Figure 16 shows the experimental results. The response time decreases drastically with OSGi based UDA. At the beginning, the delay is caused by establishing the network connection and resolving dependencies for the device access framework. As time elapses, the framework stores the data as its persistent state, and the invocation time is significantly reduced. These results demonstrate that the UDA is competent for time-critical application scenarios.

**Figure 17:**
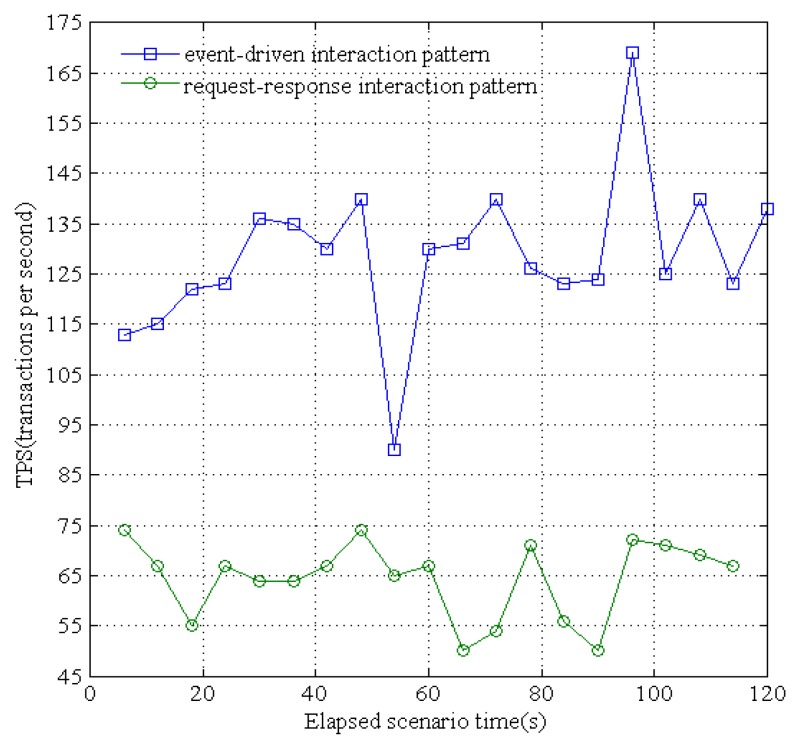
Transactions processed per second of event-driven and request-response.

**Figure 18:**
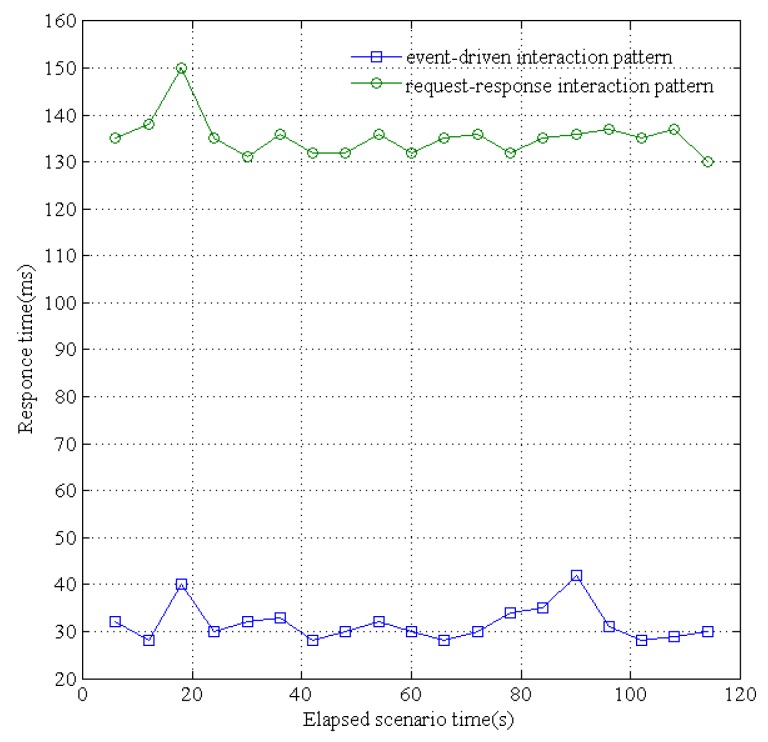
Service response time of event-driven and request-response.

The proposed resource platform supports two patterns for service interaction: event-driven (i.e., publish-subscribe) and traditional request-response. We evaluated the response times of these two patterns when concurrent sensing data are received from different underground devices. Specifically, for the convenience of receiving the sensing data and immediately dispatching them to the applications, we simulated 500 sensing devices to send concurrent data. As shown in Figure 17, the maximum processed transactions per second (TPS) of the traditional request-response pattern is 74 and the average TPS is 64.42; the maximum TPS of the publish-subscribe pattern is 167.6 and the average TPS is 130.4. In addition, as shown in Figure 18, the maximum service response time of request-response is 150 ms and the average response time is 134 ms; the maximum response time of publish-subscribe is 42 ms and the average response time is 30 ms. The publish-subscribe pattern adopts an asynchronous multicast-like communication pattern, and can dispatch the published data from waiting an acknowledgment for the subscriber. Thus, the publisher can quickly move on to the next receiver within deterministic time without any synchronous operations. Therefore, the data-centric IoT paradigm is more efficiently realized by the publish-subscribe based data distribution pattern rather than the traditional request-response pattern.

**Figure 19:**
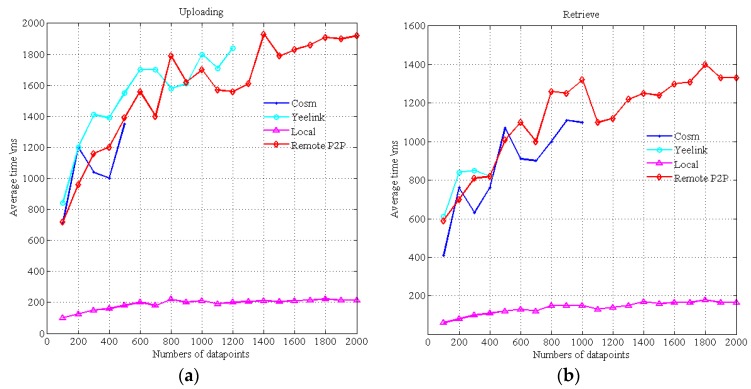
(**a**) average uploading time; (**b**) average retrieval time.

Figure 19 indicates the delay time of data uploading and retrieval. Cosm and Yeelink are well known WoT platforms that act as data broker centers for data uploading and retrieval. Local represents the local mode as discussed in Section 3. Remote P2P represents the Peer-to-Peer mode, i.e., the data provider publishes data to the remote LP in other application domain through the Internet. The data point is the data cell that contains a timestamp and a value. Yeelink limits that the minimum interval time between two requests is 10 s, and Cosm has a similar restriction. Due to these restrictions, we cannot do continuous uploading and retrieve operations. For example, we cannot test the time of continuous uploading of 1000 data points, so we only test the time of a batch operation. The maximum number of data points retrieved from Cosm in one query is 1000, and the maximum number uploads is 500. The maximum number of retrieving from Yeelink in one query is 400. We repeat 10 frequencies for each data point number and take their averages.

As shown in Figure 19a, the uploading time of Cosm, Yeelink and our Remote mode are similar. The uploading time of the local mode is stable and much smaller. The retrieval time of Cosm and Yeelink is shown in Figure 19b. Figure 19b only shows the results in the case where the data they required are already on the Cosm and Yeelink platforms. However, if the data are not on the platform (e.g., data are uploaded in pull mode instead of push mode), the time for getting the data will become longer (almost two times longer), it will bear twice the delay, i.e., from the remote data providers to Cosm and from Cosm to the consumers. However, using the P2P mode discussed in Section 3, the data are provided from the remote LPs to the consumers directly, and this will take less time. Thus, the performance of the local mode and the P2P mode both surpass Cosm and Yeelink. The results indicate that the interaction modes of this framework are more suitable for time-critical scenarios than existing commercial WoT platforms.

## 8. Conclusions

This paper proposes a WoT resource framework which provides the infrastructure for the customizable openness and sharing of users’ data and resources under the premise of guaranteeing the real-time use of their own applications. Moreover, it is able to access large scale systems of heterogeneous and legacy devices to unlock these valuable resources. The proposed framework is validated by actual system models and experimental evaluations. We do not discuss in this paper the issues of security, and privilege-based access control. Some of these topics are the subject of our m are our ongoing work. Up to now, the framework only has one single public platform, so it may cause single point of failure problems. In our future work, we envision that public platforms will be federated and have a hierarchal structure like domain name servers, distributed in the different domains of the Internet.

## Figures and Tables

**Table 1:** Summary of the DML grammar.

Type	Statement		Description
Resource declaration	@ResourceName:”ResourceURI”		Declaring a resource newly created. Generally, this is a virtual resource which will be assigned by an aggregation result of real resources.
Resource reference	“ResourceURI,Input Type, [PropertyURI]”		Define a variable by referencing a resource, ResourceURI is the URI of resource, input type indicates the type of data, including digital and analog. If the data source is entity level, PropertyURI indicates a property of this entity.
Resource validity time	[YYYY-MM-DD]T[hh:mm:ss]Z		The lifetime of resources.
Aggregation time period	[DD/hh/mm/ss]:time value		Four level time granularities of aggregation: day level, minute level, hour level and second level.
Aggregation operation	[min/max/ave/med/cur]		Operation of aggregation: minimum, maximum, average, median and latest value.
Math operations	operations	+, -, *, /, %, ^, sqrt(x)	Add, Subtract, Multiply, Divide, Modulo, Exponent, Square root
	Trigonometric Functions	sin(x), cos(x), tan(x)	Sine, Cosine, Tangent
	Inverse Trigonometric	asin(x), cos(x), atan(x)	Arcsine, Arccosine, Arctangent
	Exponentials	exp(x), sinh(x), cosh(x)	Exponential Function (e^x), Hyperbolic Sine, Hyperbolic Cosine
	Logarithms	ln(x), log2(x) log(x)	Natural (Log Base e), Binary (Log Base 2), Common (Log Base 10)
	Rounding	ceil(x), floor(x)	Ceiling, Floor
	Switch count	Switch()	Count the switching number of digital resource.
Prefixes and suffix	“prefix:[prefix]” “suffix:[ suffix]”		Attach the prefix or suffix of data, for example the unit of data can be a suffix.
Formatting	“format:[decimal places]”		This operation formats the end result by rounding the result to a specified number of digits.
